# Topology Abstraction-Based Routing Scheme for Secret-Key Provisioning in Hybrid GEO/LEO Quantum Satellite Networks

**DOI:** 10.3390/e25071047

**Published:** 2023-07-12

**Authors:** Mingxuan Guo, Yuan Cao, Jiali Zhu, Xingyu Zhou, Chunhui Zhang, Xinyi He, Xiaosong Yu, Yongli Zhao, Jie Zhang, Qin Wang

**Affiliations:** 1 School of Communications and Information Engineering, Nanjing University of Posts and Telecommunications, Nanjing 210003, China; 2 State Key Laboratory of Information Photonics and Optical Communications, Beijing University of Posts and Telecommunications, Beijing 100876, China

**Keywords:** quantum key distribution, quantum satellite network, routing scheme

## Abstract

Quantum key distribution (QKD) is a promising technique to resist the threat against quantum computers. However, the high loss of quantum signals over a long-distance optical fiber is an obstacle for QKD in the intercontinental domain. In this context, the quantum satellite network is preferred over the terrestrial quantum optical network. Due to the mobility of satellites, the satellite topology is dynamic in the quantum satellite network, which remains a challenge for routing. In hybrid geostationary-earth-orbit (GEO)/low-earth-orbit (LEO) quantum satellite networks, the lack of an efficient scheduling scheme for GEO/LEO satellites also limits the construction of quantum satellite networks. Therefore, this paper provides a topology abstraction-based routing scheme for secret-key provisioning, where the dynamic physical topology is translated into a quasi-static abstracted topology. This scheme contributes to saving the precious secret key resources. In order to improve the success probability of long-distance QKD requests, three novel resource-scheduling heuristic algorithms are proposed in hybrid GEO/LEO quantum satellite networks. Simulation results indicate that the proposed algorithms can improve the success probability of QKD requests by 47% compared to the benchmark.

## 1. Introduction

The conventional cryptographic techniques are mostly based on the mathematical complexities. They will become less reliable for critical areas in face of the threat against quantum computers [1,2]. The future-proof security techniques are gaining attention from the research community. Quantum key distribution (QKD) [3,4] has emerged for providing the unconditional security, which is based on the laws of quantum physics (e.g., Heisenberg’s uncertainty principle and no-cloning theorem) [5,6]. Compared to the conventional cryptographic techniques, it does not rely on the mathematical complexity, which can provide keys with the future-proof security level. However, due to the immaturity of existing techniques, challenges remain for practical QKD, especially in the long distance scenario.

Since the first QKD protocol, i.e., Bennett-Brassard-1984 (BB84), was developed in 1984 [7], the QKD technique over optical fibers has become more and more mature with the development of advanced QKD protocols. At the time of writing, the QKD secret key rate has achieved 115.8 Mbps over 10 km standard optical fiber in 2023 [8]. However, due to the quantum no-cloning theorem, the quantum signal cannot be amplified, which limits the QKD distance. The ultra-long-distance QKD (e.g., several thousand kilometers) over the optical fiber is extremely difficult even with current advanced protocols [9]. The trusted relay technique can be used to extend the distance of QKD, which is deployed in the practical ground QKD network. However, to achieve QKD in the intercontinental domain, a large number of trusted relays required to be deployed, which is obviously not practical for commercial use. Therefore, the optical fiber-based QKD is not an effective way to achieve a global quantum network.

The free-space QKD is expected to become a critical technique for deploying the global quantum network. Compared to the optical fiber, the quantum signal loss over the free space in a vacuum can be omitted, which makes the long-distance QKD possible. When considering the satellite-to-ground QKD, it will suffer the atmospheric loss. However, since most of the space is vacuum instead of air, the quantum signal loss over the free space is still much lower than that over an optical fiber of the same distance [10]. In 2016, a quantum low-earth-orbit (LEO) satellite, namely Micius, has successfully demonstrated the satellite-to-ground QKD [11], which proved the feasibility of the satellite QKD. Some studies have been conducted on satellite QKD strategies in the intercontinental domain. However, they mostly focused on the free-space QKD based on only a single satellite, which cannot fulfill the requirements of global scale users. Meanwhile, due to the small coverage and flyover time of a single satellite, the conventional satellite QKD strategy may bring a large secret key provisioning delay.

In order to achieve practical QKD in the global scale, it is necessary to deploy the quantum satellite network. The quantum satellite network consists of several satellites, which is currently based on the trusted-relay satellite QKD scheme [12,13,14]. Due to the mobility nature of satellites, the physical topology of the quantum satellite network is dynamic, which is a central obstacle for its construction. The topology of the fiber-based network is static; hence, the widely studied techniques (e.g., the routing scheme) for operating the fiber-based network cannot be directly applied to the quantum satellite network. Some preliminary works have been conducted; for example, Vergoossen et al. [15] analyzed the effectiveness of quantum satellites in different constellation arrangements. However, a large number of secret keys are wasted in existing routing strategies. The lack of the efficient routing scheme for key provisioning limits the construction of quantum satellite networks.

The feasibility of quantum communication between the high-orbit satellite and the ground station has been attested in [16]. The quantum GEO satellite has a larger coverage area but with a relatively low secret key rate. On the contrary, the quantum LEO satellite has a relatively high secret key rate but with the limited coverage area. However, there is a lack of efficient strategies to utilize the characteristic of both GEO and LEO in a hybrid GEO/LEO quantum satellite network, resulting in the resource wastage.

In this work, we remedy the lack of routing schemes for secret-key provisioning in hybrid GEO/LEO quantum satellite networks. We present a novel routing scheme that improves the efficiency of secret-key provisioning and propose three algorithms to fully utilize the superiorities of GEO and LEO. The main contributions of this paper are described as follows.

1. A layered architecture for quantum satellite networks is proposed, where the quantum key pool layer is decoupled from the dynamic physical topology for implementing efficient key management.
2. We present a novel topology abstraction-based routing scheme for secret-key provisioning, where the quantum key pools are utilized to construct the abstracted topology for routing over the classical channels.
3. We propose three heuristic algorithms, namely the GEO-free topology abstraction-based routing (GF-TAR) algorithm, the LEO-first topology abstraction-based routing (LF-TAR) algorithm, and the mixed satellite topology abstraction-based routing (MS-TAR) algorithm.

The rest of this paper is organized as follows. The related works are reviewed in Section 2. The network architecture is presented in Section 3. Section 4 describes the novel topology abstraction-based routing scheme for secret-key provisioning. Section 5 proposes the heuristic algorithms. Section 6 presents the simulation results and analyzes the performance of the proposed algorithms. This paper is concluded in Section 7.

## 2. Related Work

This section briefly introduces several related works, covering satellite-based QKD, quantum satellite networks, and QKD techniques in hybrid GEO/LEO scenarios.

### 2.1. Satellite-Based QKD

A detailed review can be found in [17,18,19]. Due to the transmission loss scales exponentially over the optical fiber, free-space QKD becomes an effective scheme in the context of long distances. The free-space QKD relying on the satellite is also called the satellite-based QKD. Since the first quantum satellite Micius was launched in China, many research studies have been conducted on satellite-based QKD [20,21]. The feasibility of the satellite-based QKD in daylight was proven in [22]. In [23], a time-delayed single quantum repeater node was presented for global QKD with a single satellite. However, most of the researchers focused on the QKD scheme with a single satellite, which cannot fulfill the increasing requirements of the global QKD.

### 2.2. Quantum Satellite Networks

The quantum satellite network is an effective way to achieve QKD in the intercontinental domain. Moreover, as more and more quantum satellites are planned to be launched and interconnected [14], the research on the construction and application of quantum satellite networks is inevitable. Similar to the ground optical fiber QKD network, the quantum satellites can be used as trusted relays to form a large-scale quantum network. The problem of optimizing the orbits of the quantum satellites was studied in [24]. In [25], a point-to-multipoint quantum satellite relay scheme was proposed. The modeling research of quantum satellite constellations was conducted in [26]. Most of the routing schemes in existing research studies are virtual topology-based routing schemes, which treat the dynamic physical topology as a static one over a small time interval. However, they only make efforts to overcome the mobility of the satellite instead of increasing the efficiency of secret-key provisioning.

### 2.3. QKD Techniques in Hybrid GEO/LEO Scenarios

The quantum-limited coherent measurements of optical signals were successfully sent from a GEO satellite to the ground station in 2017 [27], indicating that the GEO can implement QKD in principle. In [28], a double-layer quantum satellite network architecture was proposed, where both GEO and LEO satellites were included. In particular, the researchers are planning to launch the higher-orbit quantum satellite [12]. These works have demonstrated the value of further research on the routing and secret-key provisioning schemes in the hybrid GEO/LEO scenario. In this paper, we focus on improving the relatively low efficiency of secret-key provisioning.

## 3. Network Architecture

**Figure 1:**
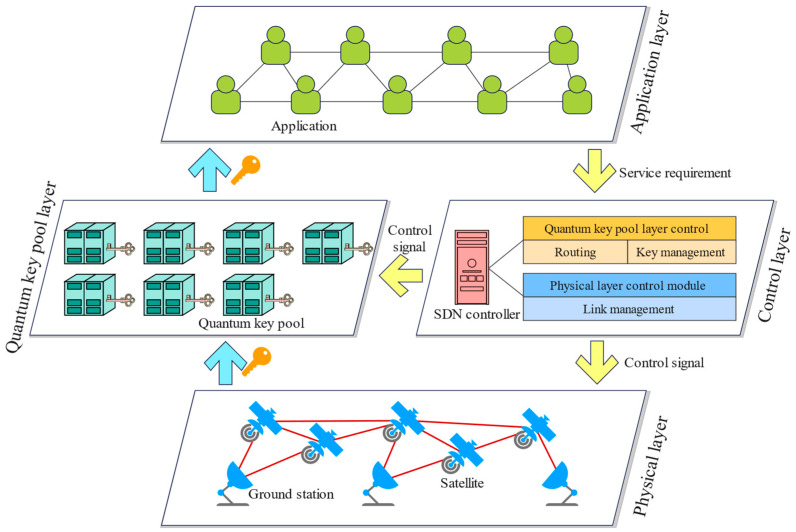
A layered architecture for quantum satellite networks.

As shown in Figure 1, the layered quantum satellite network architecture is divided into the physical layer, quantum key pool layer, control layer, and application layer.

Physical layer: It consists of the satellites and the ground stations. The satellites and ground stations are linked with each other to form the classical and quantum channels over the free space. The practical linking motion is conducted in this layer to transmit the classical signal and the quantum signal.

Quantum key pool layer: It comprises the quantum key pools (QKPs). The QKP is an effective technique to save the secret keys for timely provisioning [17], which can store the secret keys in the storage medium for reducing the key wastage. The secret keys are obtained from the physical layer to this layer for storage. This layer will be detailed in Section 4.

Control layer: It is made up with the software-defined networking (SDN) controller for the efficient resource allocation control [29,30]. The SDN controller has two control modules, namely QKP layer control module and physical layer control module. The QKP layer control module is used to route the requests over the QKP layer and manage the rest of the keys for provisioning. The physical layer control module is used to control the establishment and removal of the link between the satellite and the associated satellite/ground station.

Application layer: It consists of several applications for secret keys. This layer generates the requests for secret keys and sends the requirements to the control layer. This layer will finally obtain the secret keys from the quantum key pool layer via the key delivery application programming interface (API).

## 4. Topology Abstraction Implementation

### 4.1. Topology Abstraction-Based Routing Scheme

The satellite moves in the orbit, resulting in the dynamic change of the visibility and distance between satellites and satellites/ground stations. The satellite is controlled by the SDN controller in the control layer to establish the quantum link with other sites (including satellites and ground stations) based on specific algorithms, for example, establishing the quantum link with the closest site. The generated secret keys will be provided for the corresponding QKP in the QKP layer. Due the periodicity of the relationship between satellites and ground stations, the arrangement of the establishment for the quantum link between two sites is also periodic based on the specific algorithm. Once a pair of sites has established a quantum link in a period, there exists a corresponding QKP in the QKP layer. Meanwhile, each site is abstracted as a fixed node in the QKP layer. If there is a QKP between a pair of abstracted nodes, then the two nodes are connected on the abstracted topology, since the corresponding nodes can share secret keys with each other relying on a QKP. The global keys can be relayed by the shared keys even if there is no quantum link of the corresponding site pair at that time, which will be detailed in Section 4.2. As exemplified in Figure 2a, the dynamic physical topology in the physical layer is translated into a fixed abstracted topology in the QKP layer. For example, although LEO-2 and LEO-3 are unconnected in the physical layer now, they will be connected in other time slots in a period. Hence, there is a link between Node-2 and Node-3 on the abstracted topology. Other links, e.g., the link between Node-1 and Node-4 as well as the link between Node-1 and Node-3, can also be attributed to this reason. As depicted in Figure 2a, a QKD request from Ground station-a to Ground station-c is generated, and we route for this request on the abstracted topology to obtain the QKD relaying path (i.e., the red lines in Figure 2a). The QKD relaying path refers to the path of relaying the global secret keys hop by hop with the exclusive OR operation.

### 4.2. Topology Abstraction-Based Key Relaying Strategy

After obtaining the QKD relaying path from the routing process on the abstracted topology, we use the classic channel (e.g., laser channel) to relay the global secret keys following the QKD relaying path. Due to the relatively high data rate over the classic channel, there can be a large number of classic channels between two sites with the time-division-multiplexing (TDM) technique. In the hybrid GEO/LEO network, due to the large coverage of the GEO satellite, there commonly exist classic channels to send the encrypted global secret keys to the corresponding site (in theory, three GEOs can fully cover the entire earth). Even if a GEO does not exist in the network, due to the large number of LEOs in most of the satellite constellations, it still can find a path to send the encrypted key to any site in most cases. As shown in Figure 2a, the QKD relaying path is from Node-a to Node-2, then, it is from Node-2 to Node-3, and finally, it is from Node-3 to Node-c. Since LEO-2 and LEO-3 cannot establish the link at this time (they are not visible to each other), we first send the encrypted global secret key (i.e., key_a-2_ ⊕ key_2-3_) from LEO-2 to GEO; then, the GEO forwards this key to LEO-3 to decrypt for the global secret key (i.e., key_a-2_), as shown in Figure 2b. Finally, LEO-3 send the encrypted global secret key (i.e., key_a-2_ ⊕ key_c-3_) to Ground station-c to decrypt for the global secret key; then, Ground station-a and Ground station-c can successfully share the secret key.

## 5. Heuristic Algorithm Design

Based on the topology abstraction-based routing scheme, in this section, we design three heuristic algorithms, i.e., GF-TAR, LF-TAR, and MS-TAR algorithms, to efficiently allocate the time and secret key resources in hybrid GEO/LEO quantum satellite networks. The notations and their definitions used in this paper are listed in Table 1.

The success probability (*SPT*(*t_i_*)) of the key service requests in the time slot *t_i_* can be defined as follows.

(1) $$SPT(t_{i})=\frac{N_{r}(t_{i})}{\left|R(t_{i})\right|}$$

The success probability (*SP*) of the key service requests within the simulation time can be determined as follows (*T* is defined as the total simulation time).

(2) $$SP=\frac{\sum_{i=1}^{T/\Delta }SPT(t_{i})}{T/\Delta }$$

The average number of relaying hops (*AHT*(*t_i_*)) for the successful key service requests in the time slot *t_i_* can be expressed as follows.

(3) $$AHT(t_{i})=\frac{N_{h}(t_{i})}{N_{r}(t_{i})}$$

The average number of relaying hops (*AH*) for the successful key service requests within the simulation time can be calculated as follows.

(4) $$AH=\frac{\sum_{i=1}^{T/\Delta }AHT(t_{i})}{T/\Delta }$$

The total number of secret keys (*TKT*(*t_i_*)) produced in the time slot *t_i_* can be described as follows.

(5) $$TKT(t_{i})=\phi_{l}(t_{i})+\phi_{g}(t_{i})$$

The total number of secret keys (*TK*) produced within the simulation time can be calculated as follows.

(6) $$TK=\sum_{i=1}^{T/\Delta }TKT(t_{i})$$

### 5.1. GEO-Free Topology Abstraction-Based Routing Algorithm

The process of the GEO-free topology abstraction-based routing algorithm (GF-TAR) is detailed in Algorithm 1. This algorithm abstracts the topology of LEO satellites and ground stations for routing, where the LEO is used for QKD and the GEO is used for assisting the transmission of classic signals of the encrypted global keys. Line 1 is used for the initialization of the elements. Under the limitation *l*_g_, we make the LEO satellites establish the quantum channels with several of the closest ground stations in lines 2–11 to produce the max number of secret keys to the QKPs. In this process, we use the First-Fit (FF) algorithm to select the ground stations to be connected, which is efficient and normally used in resource allocation. In lines 12–20, we conduct the similar operation to establish the quantum channels between LEOs under the limitation *l*_b_. The secret keys produced in this time slot *t_i_* are provided to corresponding QKPs in lines 22–24. We update the abstracted topology according to the rest of the key resources on the link in lines 27–32. If the number of keys on the link cannot reach the minimum value of the set of secret keys required for the request *K*_s_, we cut off this link on the abstracted topology until new enough keys are supplied to the QKP on this link. We route for the request on the abstracted topology to obtain the QKD relaying path in line 33. The minimum value of the rest of the key resources on the links along the relaying path is obtained in lines 35–40. In lines 41–47, the comparison between the required key resources and the minimum value of the rest of the key resources on the relaying path is conducted. If the required number of key resources is smaller, we allocate the key resources to implement the request; otherwise, the request fails.
**Algorithm 1**: GF-TAR algorithm.
**Input:** *K*(*t_i_*), *R*(*t_i_*), *V*_g_, *V*_l_, *P*(*t_i_*)**Output:** *SPT*(*t_i_*), *AHT*(*t_i_*), *TKT*(*t_i_*), *P*(*t_i_*_+1_) and solutions for the requests1:Initialize *SPT*(*t_i_*) ← 0, *AHT*(*t_i_*) ← 0, *c*(*m*, *n, t_i_*) ← 0, vs. ← *V*_g_ ∪ *V*_l_;2:**for** *n* ∈ *V*_l_ **do**3: **if** $\sum_{m\in V_{g}}\left(v(m,n,t_{i})>0\right)>l_{g}$ **do**4:  *χ* ← *l*_g_;5: **else**
6:  $\chi \leftarrow \sum_{m\in V_{g}}\left(v(m,n,t_{i})>0\right)$;7: **end if**
8: Find the *χ* largest element in *v*(*m*, *n*, *t_i_*), *m* ∈ *V*_g_, and record their *m* to the set Λ;9: **for** *m* ∈ Λ **do**10:  *c*(*m*, *n*, *t_i_*) ← 1;11: **end for**
12: **if** $\sum_{m\in V_{l}}\left(v(m,n,t_{i})>0\right)>l_{b}$ **do**13:  $\chi \leftarrow l_{b}-\sum_{m\in V_{l}}c(m,n,t_{i})$;14: **else**15:  $\chi \leftarrow \sum_{m\in V_{l}}\left(v(m,n,t)>0\right)-\sum_{m\in V_{l}}c(m,n,t_{i})$;16: **end if**17: $\mathrm{Find}\;\mathrm{the}\;\chi \;\mathrm{largest}\;\mathrm{element}\;\mathrm{in}v(m,n,t_{i})\cdot (1-c(m,n,t_{i}))$, *m* ∈ *V*_l_, and record their *m* to the set Λ;18: **for**
*m* ∈ Λ **do**19:  *c*(*m*, *n, t_i_*) ← 1;20: **end for**21:**end for**22:**for** $m\in V_{s},n\in V_{s}$ **do**23: $p(m,n,t_{i})\leftarrow p(m,n,t_{i})+c(m,n,t_{i})\cdot v(m,n,t_{i})\cdot \Delta$;24:**end for**25:$TKT(t_{i})\leftarrow \sum_{m\in V_{s}}\sum_{n\in V_{s}}c(m,n,t_{i})\cdot v(m,n,t_{i})\cdot \Delta$;26:**for** *r*(*s_r_, d_r_, k_r_*, *t_i_*) ∈ *R*(*t_i_*) **do**27: *flag* ← 0, *E*_s_ ← ∅;28:  **for**
$m\in V_{s},n\in V_{s}$ **do**29:   **if**
$p(m,n,t_{i})\geq \min(K_{s})$ **do**30:    $E_{s}\leftarrow E_{s}\cup \{(m,n)\}$;31:   **end if**32:  **end for**33:  *P*_d_ ← routing from *s_r_* to *d_r_* in *G*_s_(*V*_s_, *E*_s_) with Dijkstra algorithm;34:  **if**
$P_{d}\neq \emptyset$ **do**35:   *μ* ← ∞;36:   **for** (*m*, *n*) ∈ *P*_d_ **do**37:    **if**
*p*(*m*, *n*) <*μ* **do**38:     *μ* ← *p*(*m*, *n*);39:    **end if**40:   **end for**41:   **if**
*μ* >*k_r_* **do**42:    $SPT(t_{i})\leftarrow SPT(t_{i})+1,AHT(t_{i})\leftarrow AHT(t_{i})+|P_{d}|,flag\leftarrow 1$;43:    Record the solutions for the request *r*(*s_r_, d_r_, k_r_*, *t_i_*);44:    **for** (*m*, *n*) ∈ *P*_d_ **do**45:     $p(m,n,t_{i})\leftarrow p(m,n,t_{i})-k_{r}$;46:    **end for**47:   **end if**48:  **end if**49:**end for**50:**return** $AHT(t_{i})\leftarrow AHT(t_{i})/SPT(t_{i}),SPT(t_{i})\leftarrow SPT(t_{i})/|R(t_{i})|,P(t_{i+1})\leftarrow P(t_{i})$, *TKT*(*t_i_*), and solutions for the requests set *R*(*t_i_*).

The time complexity of the GF-TAR algorithm is evaluated as follows. The worst-case time complexities in lines 2–21 and 22–24 are $O((l_{g}+l_{b})\cdot |V_{l}|)$ and $O(|V_{g}\cup V_{l}{|}^{2})$, respectively. The time complexity in lines 28–48 is $O(|V_{g}\cup V_{l}{|}^{2})$. The overall complexity in lines 26–49 is $O(|R(t_{i})|\cdot |V_{g}\cup V_{l}{|}^{2})$. Hence, the worst-case time complexity of the GF-TAR algorithm in a time slot is $O(|R(t_{i})|\cdot |V_{g}\cup V_{l}{|}^{2}+(l_{g}+l_{b})\cdot |V_{l}|)$.

### 5.2. LEO-First Topology Abstraction-Based Routing Algorithm

The process of the LEO-first topology abstraction-based routing (LF-TAR) algorithm is depicted in Algorithm 2. In the LF-TAR algorithm, the requests are firstly routed on the abstracted topology of LEOs and ground stations. If the rest of the key resources on the abstracted topology cannot fulfill the requirements of the request, then we utilize the GEOs to establish corresponding quantum channels on the physical topology for QKD to fulfill the requirements. The GEO-based QKD is used as an alternative plan for the LEO-based QKD. The abstracted topology of GEOs and ground stations is not established, while the GEOs establish the quantum channels for QKD according to the requests. Lines 1–2 serve as the same function in the GF-TAR algorithm. The time resources of the links on the physical topology of GEOs and ground stations are initialized in line 3. It should be noted that one GEO can establish *l*_h_ quantum channels to the ground stations. Hence, the time resources of links from the GEO to the ground station is Δ·*l*_h_ in numerical terms. Line 5 serves as the same function of the GF-TAR algorithm, i.e., to firstly try to fulfill the requirements of the requests on the abstracted topology of LEOs and ground stations. If it failed to fulfill the requirements in line 5, we try to implement the requests by the GEO-based QKD. In lines 6–16, we update the physical topology of GEOs and ground stations based on the rest of the time resources of the links. If the rest of the time resources cannot fulfill the minimum requirements of the set of secret keys required, we remove this link from the physical topology. The QKD relaying path is obtained by routing on the physical topology *G*_c_(*V*_c_, *E*_c_) of GEOs and ground stations in line 17. If the time resources are sufficient on the links along the QKD relaying path, we allocate the time resources to the request in lines 18–32.
**Algorithm 2**: LF-TAR algorithm.
**Input:** *K*(*t_i_*), *R*(*t_i_*), *V*_g_, *V*_l_, *V*_h_, *P*(*t_i_*), *E*_ε_, *E*_Ω_**Output:** *SPT*(*t_i_*), *AHT*(*t_i_*), *TKT*(*t_i_*), *P*(*t_i_*_+1_), and solutions for the requests1:Initialize *SPT*(*t_i_*)← 0, *AHT*(*t_i_*) ← 0, *c*(*m*, *n, t_i_*) ← 0, vs. ← *V*_g_ ∪ *V*_l_, *V*_c_ ← *V*_g_ ∪ *V*_h_;2:Execute lines 2–25 in GF-TAR algorithm;3:$f_{\alpha }(m,n,t_{i})\leftarrow \Delta \cdot l_{h},f_{\beta }(m,n,t_{i})\leftarrow \Delta ,E_{c}\leftarrow E_{\varepsilon }\cup E_{\Omega }$;4:**for** *r*(*s_r_, d_r_, k_r_*, *t_i_*) ∈ *R*(*t_i_*) **do**5: Execute lines 27–48 in GF-TAR algorithm;6: **if** flag = 0 **then**7:  **for** (*m*, *n*) ∈ *E*_c_ **do**8:   **if** (*m*, *n*) ∈ *E*_Ω_ **do**9:    **if** 
$v(m,n)\cdot f_{\beta }(m,n,t_{i})<\min(K_{s})$ **do**
10:     $E_{c}\leftarrow E_{c}-\{(m,n)\}$;11:    **end if**
12:   **else**
13:    **if** $v(m,n)\cdot f_{\alpha }(m,n,t_{i})<\min(K_{s})$ **do**
14:     $E_{c}\leftarrow E_{c}-\{(m,n)\}$;15:    **end if**
16:  **end for**
17:  *P*_d_ ← routing from *s_r_* to *d_r_* in *G*_c_(*V*_c_, *E*_c_) with Dijkstra algorithm;18:  **if** $P_{d}\neq \emptyset$ **do**19:   *ξ* ← 1;20:   **for** (*m*, *n*) ∈ *P*_d_ **do**21:    **if** 
$(m,n)\in E_{\varepsilon }\&\;\&(f_{\alpha }(m,n,t_{i})-k_{r}/v(m,n,t_{i}))<0$ **do**
22:     *ξ* ← 0;23:    **elseif** 
$(m,n)\in E_{\Omega }\&\;\&(f_{\beta }(m,n,t_{i})-k_{r}/v(m,n,t_{i}))<0$ **do**
24:     *ξ* ← 0;25:    **end if**
26:   **end for**
27:   **if**
*ξ* = 1 **do**28:    $TKT(t_{i})\leftarrow TKT(t_{i})+k_{r},SPT(t_{i})\leftarrow SPT(t_{i})+1,AHT(t_{i})\leftarrow AHT(t_{i})+|P_{d}|$, and update the *f*_α_(*m*, *n, t_i_*) and *f*_β_(*m*, *n, t_i_*) to all the (*m*, *n*) ∈ *P*_d_;29:    Record the solutions for the request *r*(*s_r_, d_r_, k_r_*, *t_i_*);30:   **end if**
31:  **end if**
32: **end if**
33:**end for**34:**return** $AHT(t_{i})\leftarrow AHT(t_{i})/SPT(t_{i}),SPT(t_{i})\leftarrow SPT(t_{i})/|R(t_{i})|,P(t_{i+1})\leftarrow P(t_{i})$, *TKT*(*t_i_*), and solutions for the requests set *R*(*t_i_*).

The time complexities of lines 2 and 5 have been evaluated in the GF-TAR algorithm. The time complexity in lines 7–31 is $O(|V_{g}\cup V_{h}{|}^{2})$. The overall time complexity of the LF-TAR algorithm in a time slot is $O(|R(t_{i})|\cdot (|V_{g}\cup V_{l}{|}^{2}+|V_{g}\cup V_{h}{|}^{2})+(l_{g}+l_{b})\cdot |V_{l}|)$.

### 5.3. Mixed Satellite Topology Abstraction-Based Routing Algorithm

In Algorithm 3, the detail process of the mixed satellite topology abstraction-based routing (MS-TAR) algorithm is depicted. In this algorithm, the GEOs are also included in the abstracted topology with the LEOs and ground stations for routing. The GEO plays the same role as the LEO on the abstracted topology. Lines 1–2 perform the same function in the GF-TAR algorithm. In lines 3–5, we establish the quantum channels between GEOs. The GEOs are connected with each other due to the large coverage of GEOs. In lines 6–22, the GEO establishes the quantum channels for QKD to several closest sites (including LEOs and ground stations) under the limitation of *l*_h_. Line 23 performs the same operations in the GF-TAR algorithm to update the QKPs and route for the requests on the abstracted topology.
**Algorithm 3**: MS-TAR algorithm.
**Input:** *K*(*t_i_*), *R*(*t_i_*), *V*_g_, *V*_l_, *V*_h_, *P*(*t_i_*), *E*_τ_**Output:** *SPT*(*t_i_*), *AHT*(*t_i_*), *TKT*(*t_i_*), *P*(*t_i_*_+1_), and solutions for the requests1:Initialize *SPT*(*t_i_*)← 0, *AHT*(*t_i_*) ← 0, *c*(*m*, *n, t_i_*) ← 0, vs. ← *V*_g_ ∪ *V*_l_ ∪ *V*_h_;2:Execute lines 2–21 in GF-TAR algorithm;3:**for** $m\in V_{h},n\in V_{h},m\neq n$ **do**4: *c*(*m*, *n*, *t_i_*) ← 1;5:**end for**6:**for** *n* ∈ *V*_h_ **do**7: **if** $\sum_{m\in V_{g}\cup V_{l}}\left(v(m,n,t_{i})>0\right)-\sum_{(m,n)\in E_{\tau }}c(m,n,t_{i})>l_{h}$ **do**8:  *χ* ← *l*_h_;9: **else**
10:  $\chi \leftarrow \sum_{m\in V_{g}\cup V_{l}}\left(v(m,n,t_{i})>0\right)-\sum_{(m,n)\in E_{\tau }}c(m,n,t_{i})>l_{h}$;11: **end if**
12: Ψ ←∅;13: **for**
*m* ∈ *V*_g_ ∪ *V*_l_ **do**14:  **if** $∼((m,n)\in E_{\tau }\&\;\&\sum_{ο\in V_{h}}c(m,ο,t_{i})>0)$ **do**15:   $\Psi \leftarrow \Psi \cup \{v(m,n,t_{i})\}$;16:  **end if**
17: **end for**
18: Find the *χ* largest *v*(*m*, *n*, *t_i_*) in the set Ψ, and record their *m* to the set Λ;19: **for** *m* ∈ Λ **do**20:  *c*(*m*, *n*, *t_i_*) ← 1;21: **end for**
22:**end for**23:Execute lines 22–49 in GF-TAR algorithm;24:**return** $AHT(t_{i})\leftarrow AHT(t_{i})/SPT(t_{i}),SPT(t_{i})\leftarrow SPT(t_{i})/|R(t_{i})|,P(t_{i+1})\leftarrow P(t_{i})$, *TKT*(*t_i_*), and solutions for the requests set *R*(*t_i_*).

The worst-case time complexities in lines 2 and 3–22 are $O((l_{g}+l_{b})\cdot |V_{l}|)$ and $O((l_{h}+|V_{g}\cup V_{l}|)\cdot |V_{h}|)$, respectively. The time complexity in line 23 is $O(|R(t_{i})|\cdot |V_{g}\cup V_{l}\cup V_{h}{|}^{2})$. The overall time complexity of the MS-TAR algorithm in a time slot is $O(|R(t_{i})|\cdot |V_{g}\cup V_{l}\cup V_{h}{|}^{2}+(l_{g}+l_{b})\cdot |V_{l}|+(l_{h}+|V_{g}\cup V_{l}|)\cdot |V_{h}|)$.

## 6. Evaluation and Analysis

**Figure 3:**
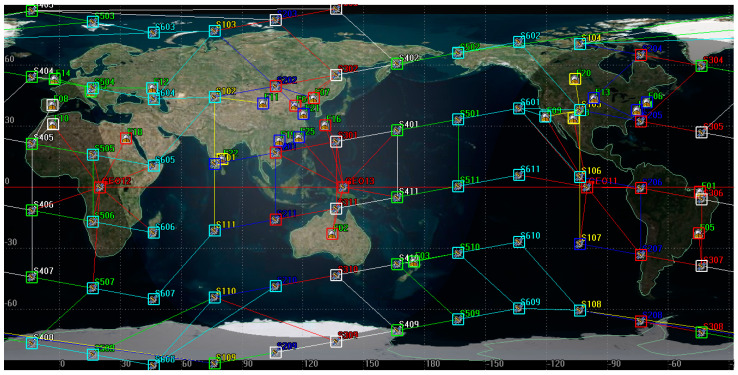
An example of the 6-plane Star topology with 25 ground stations, 3 GEOs, and a Walker Star constellation containing 66 LEOs.

To evaluate the performance of the GF-TAR, LF-TAR, and MS-TAR algorithms, we perform the simulations with four satellite network topologies. All the topologies consist of 25 ground stations randomly chosen from the major ground stations around the world, as shown in Figure 3. Three GEOs in the Walker constellation with one orbital plane are also included in all the topologies. In the six-plane Star topology and seven-plane Star topology, we construct a Walker Star constellation with six and seven orbital planes, respectively. In the six-plane Delta topology and seven-plane Delta topology, we construct a Walker Delta constellation with six and seven orbital planes, respectively. The Walker Star and Walker Delta are two typical constellations [25,31]. Each of the LEO orbital planes has 11 LEO satellites. Figure 3 shows the distribution of ground stations and satellites on the six-plane Star topology as an example of topologies. The LEO satellite has an orbit altitude of 500 km, an inclination of 90 degrees, and an orbital period of 5677 s. The GEO satellite has an orbit altitude of 35788.1 km, an inclination of 0 degrees, and an orbital period of 86170.5 s (approximately equal to 24 h). Due to the ground coverage time of LEO being commonly up to 5 min, we set the duration of the physical discrete topology to 1 min (i.e., Δ is equal to 1 min). We set the simulation time *T* to 48 h and then obtain 2881 physical topologies for different moments. We jointly use the STK 11.6 and MATLAB 2018b to obtain the related access and distance information of the satellites as well as process the dates to obtain the physical topology matrixes. Then, we use the MATLAB for the simulations of the proposed algorithms and the benchmark algorithms. The simulations are performed on a computer with 2.3 GHz Intel Core i7–10875H CPU and 16 GB RAM.

In the simulation, each LEO can create quantum links to four ground stations (i.e., *l*_g_ is equal to four) and each LEO can establish quantum links with four LEOs (i.e., *l*_b_ is equal to four). Each GEO can establish quantum links to eight LEOs or ground stations (i.e., *l*_h_ is equal to eight) and each LEO can establish the quantum link with one GEO. The keys required for the request *k_r_* are randomly chosen from the set *K*_s_ = {0.8*ϖ*, *ϖ*, 1.2*ϖ*}. The QKD key rates are set according to the key rate data in [11,22]. Given that there is little research on the hybrid GEO/LEO quantum satellite network, we use four simple benchmark algorithms in this paper. The GEO-free benchmark with non-adaptive topology (Benchmark-GFN) and GEO-free benchmark with topology updating (Benchmark-GFU) use only LEOs for QKD. The LEO-first benchmark with non-adaptive topology (Benchmark-LFN) and LEO-first benchmark with topology updating (Benchmark-LFU) use both GEOs and LEOs for QKD, where the GEO-based QKD is used as an alternative plan for the LEO-based QKD. The Benchmark-GFN and Benchmark-LFN route on the physical topology without updating according to the rest of the time resources on the link. The Benchmark-GFU and Benchmark-LFU route on the physical topology with updating according to the rest of the time resources on the link. In Section 6.1, we will analyze the success probability and the max traffic supported on different topologies. In Section 6.2, we will compare the average number of relaying hops for the successful requests. The analysis of the total number of secret keys produced is conducted in Section 6.3.

### 6.1. Performance Evaluation on the Success Probability and the Max Traffic Supported

**Figure 4:**
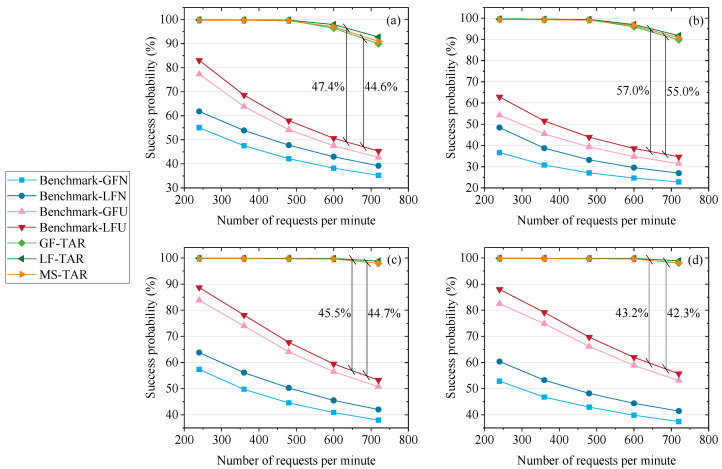
Success probability versus number of requests per minute on the (**a**) 6-plane Star topology, (**b**) 6-plane Delta topology, (**c**) 7-plane Star topology and (**d**) 7-plane Delta topology.

The results of success probability (*SP*) versus the number of requests per minute on different topologies are depicted in Figure 4a–d. We set the average keys for one request (*ϖ*) as 100 kbits. The *SP* on the topology with seven-plane constellation is higher than that on the topology with six-plane constellation, since there are more satellites for QKD on the topology with seven-plane constellation. Obviously, the proposed algorithms perform better than the benchmark algorithms in terms of the success probability. For example, the GF-TAR and LF-TAR algorithms increase the *SP* by 44.6% and 47.4%, respectively, compared to the Benchmark-LFU on the six-plane Star topology. This is because the topology abstraction-based routing scheme can fully save the secret keys in the QKP of the link on the abstracted topology. The quantum links are established for immediate requests in benchmark algorithms, and the free time resources are wasted due to the lack of key management. Meanwhile, the proposed algorithms establish the shortest quantum link to ensure the largest number of keys provided to QKPs, which contributes to the success of requests. In addition, on the abstracted topology, the links are increased compared to the physical topology. Hence, the number of relaying hops will decrease, and fewer keys are wasted due to the relaying operation. It can also create more possible paths for the request to increase the *SP*.

The Benchmark-GFU and Benchmark-LFU have a higher *SP* than the Benchmark-GFN and Benchmark-LFN, respectively. This is because the former two benchmark algorithms update the physical topology according to the rest time resources, which can initially prevent the QKD relaying path including some links lacking resources. The Benchmark-LFN and Benchmark-LFU perform better than the Benchmark-GFN and Benchmark-GFU, respectively, since the GEO-based QKD is utilized in the former two benchmark algorithms. Furthermore, the LF-TAR and MS-TAR algorithms perform better than the GF-TAR algorithm, since the GF-TAR does not utilize the GEO satellites for QKD, which decreases the number of secret keys produced. The LF-TAR has a better performance than the MS-TAR in terms of the *SP*. This is because in the MS-TAR algorithm, the GEO is also included in the abstracted topology, which serves as the same role with the LEO. In this scheme, the GEO actually serves as an LEO with a low secret key rate, which cannot utilize the characteristic of the GEO that has a large coverage. Due to the large coverage of GEO, it can establish quantum links with many sites. However, owing to the limitation of quantum transmitters/receivers that can be carried by a GEO satellite, the number of quantum links established cannot exceed *l*_h_. If we fix the GEO to link with the closest sites, it is not efficient to improve the *SP*. Furthermore, since the number of secret keys produced by the GEO is relatively low, it does not urgently need the secret key management based on the topology abstraction scheme. In the LF-TAR algorithm, we make the GEO-based QKD as an alternative plan for the LEO-based QKD. It can accurately fulfill the requirements of the requests failed to be fulfilled by the LEO-based QKD. This can effectively utilize the characteristic of GEO that has a large coverage.

**Figure 5:**
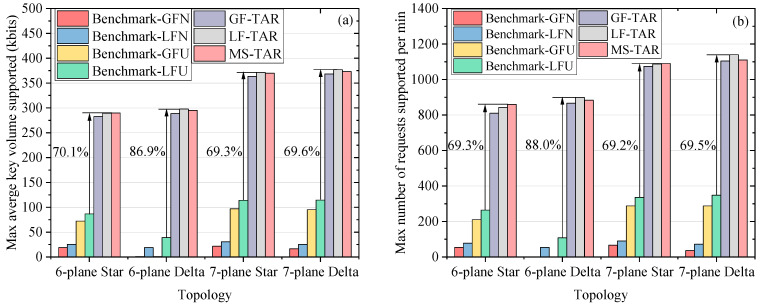
(**a**) Max average key volume supported and (**b**) max number of requests supported per minute for different algorithms on the four satellite network topologies.

We also evaluate the max traffic supported on different topologies. Figure 5a shows the results of the max average key volume supported (*MAK*) for different algorithms on the four topologies, where the number of requests per minute is fixed as 300 (i.e., |*R*(*t_i_*)| = 300) and the success probability is required to exceed 80%. The *MAK* refers to the max average keys for one request, which can be supported by the resources. The results of the max number of requests supported per minute (*MNR*) for different algorithms on the four topologies are depicted in Figure 5b, where the *ϖ* is fixed as 100 kbits and the success probability is required to exceed 80%. The proposed algorithms are superior to the benchmark algorithms in terms of *MAK* and *MNR*. For example, the MS-TAR algorithm can increase the *MAK* by 70.1% compared to Benchmark-LFU on the six-plane Star topology. The LF-TAR algorithm can increase the *MNR* by 69.5% compared to the Benchmark-LFU on the eight-plane Delta topology. This is because the proposed algorithms can save the secret keys in the QKPs of links on the abstracted topology as well as can reduce the key wastage both in the key processing stage and in the key relaying stage. It should be noted that the *MAK* and *MNR* are equal to 0 for Benchmark-GFN and Benchmark-GFU on the six-plane Delta topology. The reason is that the routing success probability is less than 80% on the six-plane Delta physical topology without GEOs, where some ground stations cannot be linked with quantum channels at certain moments. The proposed algorithms will not face this problem due to the existence of the abstracted topology and the utilization of the classical channels. The proposed algorithms have a stronger improvement effect on the Delta constellation compared to the benchmark algorithms. This is because the Delta constellation is poor in the coverage of the selected ground stations. In this situation, the benchmark algorithms are extremely weak, because it cannot save the keys in QKPs of links on the abstracted topology, resulting in the failure of requests for the ground stations connected to few or no satellites at certain moments. The LF-TAR algorithm performs similarly to the MS-TAR algorithm in terms of the *MAK* and *MNR*, and they perform better than the GF-TAR algorithm due to the existence of GEO-based QKD.

Hence, our proposed algorithms perform superior to the benchmark algorithms in terms of the success probability and the max traffic supported. The LF-TAR algorithm is the best compared to other proposed algorithms, which can fully utilize the characteristics of both LEO and GEO.

### 6.2. Performance Evaluation on the Average Number of Relaying Hops

**Figure 6:**
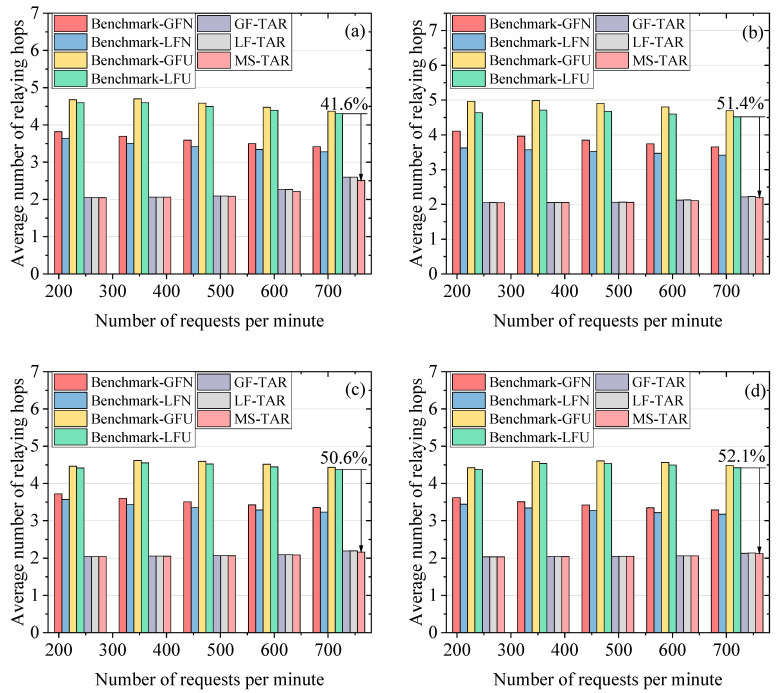
Average number of relaying hops versus number of requests per minute on the (**a**) 6-plane Star topology, (**b**) 6-plane Delta topology, (**c**) 7-plane Star topology and (**d**) 7-plane Delta topology.

In Figure 6a–d, the results of the average number of relaying hops (*AH*) versus the number of requests per minute on different topologies are depicted, where the *ϖ* is set as 100 kbits. The proposed algorithms achieve a lower average number of relaying hops compared to the benchmark algorithms, which can save the precious key resources by decreasing the key wastage for relaying. This is because our proposed algorithms implement routing on the abstracted topology, which has far more links than the physical topology. This can be beneficial to find the shortest path with a lower number of hops for the request. The *AH* values for the Benchmark-LFN and Benchmark-LFU are relatively lower than those for the Benchmark-GFN and Benchmark-GFU, respectively. This is because the number of relaying hops for GEO-based QKD is normally two or three, which can pull down the value of *AH*. The Benchmark-GFU and Benchmark-LFU have relatively higher *AH* values than the Benchmark-GFN and Benchmark-LFN, respectively, since the topology-updating process can increase the success probability at the cost of increasing the number of relaying hops. The GF-TAR algorithm and LF-TAR algorithm show similar performance in terms of the *AH*, since the number of relaying hops for GEO-based QKD is normally two or three, which can hardly influence TAR algorithms, where the *AH* approaches two. Among the proposed algorithms, the MS-TAR algorithm shows the best *AH* performance. This is because the abstracted topology has more links for the MS-TAR algorithm compared to that for the GF-TAR and LF-TAR algorithms thanks to the adding of GEO nodes and related links to the abstracted topology for the MS-TAR algorithm.

### 6.3. Performance Evaluation on the Total Number of Secret Keys Produced

**Figure 7:**
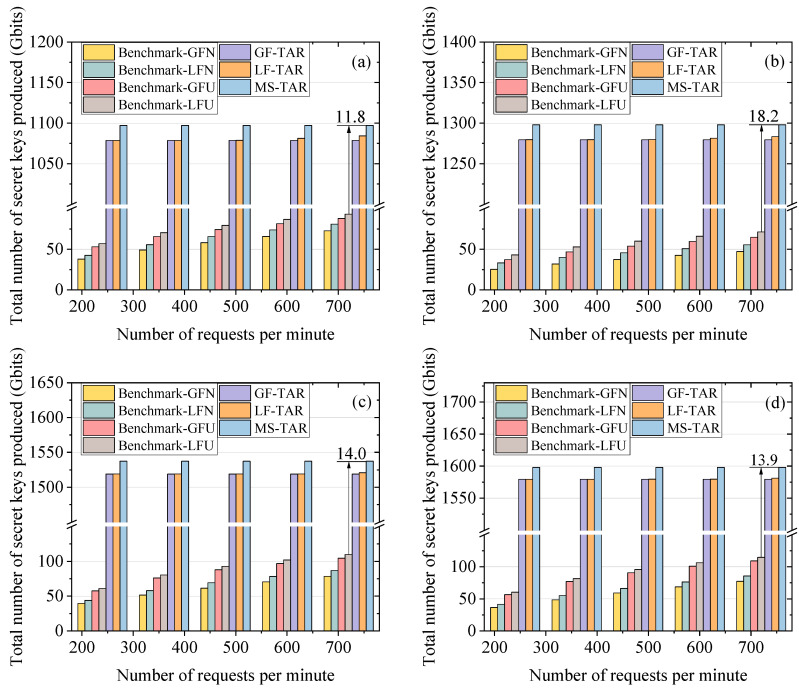
Total number of secret keys produced versus number of requests per minute on the (**a**) 6-plane Star topology, (**b**) 6-plane Delta topology, (**c**) 7-plane Star topology and (**d**) 7-plane Delta topology.

Figure 7 demonstrates the total number of secret keys produced (*TK*) versus the number of requests per minute on different topologies, where our proposed algorithms show much higher value than the benchmark algorithms. From Figure 7a, we can see that the *TK* for the MS-TAR algorithm is 11.8 times larger than that for the Benchmark-LFU. The reason is that the topology abstraction-based routing scheme can fully utilize the time to produce and inject keys into the QKPs without wastage. Meanwhile, it establishes the quantum link for each pair of sites with the lowest distance to generate the highest key rate. We can observe that the relationship of *TK* for benchmark algorithms is the same with that of *SP* for benchmark algorithms. This is because the keys in the benchmark algorithms are produced based on the requirements of the requests. Hence, the higher the number of successful requests for the benchmark algorithm, the more keys will be produced throughout the entire process. In addition, the LF-TAR and MS-TAR algorithms can produce more keys than the GF-TAR algorithm, which can be attributed to the extra keys produced by GEO-based QKD in the former two algorithms. It can also be observed that the *TK* of MS-TAR is higher than that of LF-TAR. This is due to the fact that the GEOs are linked to the closest sites for achieving a higher key rate in the MS-TAR algorithm. However, in the LF-TAR algorithm, the GEO-based QKD is used as an alternative plan to produce keys for the requirements of the requests which are not fulfilled by LEO-based QKD. The time resource wastage of the GEO satellites for QKD is the major reason for a relatively lower *TK* of the LF-TAR algorithm compared to that of the MS-TAR algorithm.

## 7. Conclusions

This paper proposes a novel topology abstraction-based routing scheme, which can abstract the dynamic physical topology into a quasi-static abstracted topology for achieving a high success probability of requests. We present three heuristic algorithms based on the topology abstraction scheme, namely GF-TAR, LF-TAR, and MS-TAR. Simulation results indicate that our proposed algorithms have a better performance in terms of the success probability, the max traffic supported, the average number of relaying hops, and the total number of keys produced. The LF-TAR algorithm stands out in the hybrid GEO/LEO quantum satellite network scenario, which exhibits the best performance by collaborating GEOs with LEOs relying on the proposed routing scheme. In future work, we will conduct more research on a hybrid GEO/LEO quantum satellites network to facilitate their construction in the real world.

## Figures and Tables

**Figure 2:**
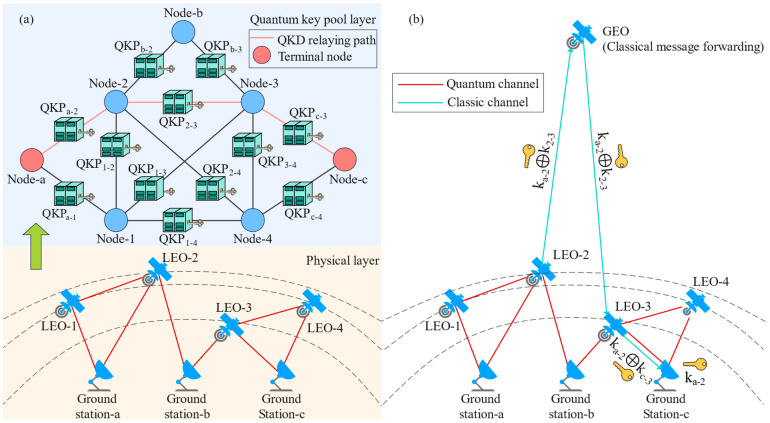
Illustration of topology abstraction-based (**a**) routing scheme and (**b**) key relaying strategy.

**Table 1:** Notations and Definitions.

Notation	Definition
*G*_s_(*V*_s_, *E*_s_)	Abstracted topology
*G*_c_(*V*_c_, *E*_c_)	Physical topology of GEOs and ground stations
*V*_s_	Set of nodes on the abstracted topology
*E*_s_	Set of links on the abstracted topology
*V*_c_	Set of nodes on the physical topology of GEOs and ground stations
*E*_c_	Set of links on the physical topology of GEOs and ground stations
*V*_g_	Set of nodes of the ground stations
*V*_l_	Set of nodes of the LEO satellites
*V*_h_	Set of nodes of the GEO satellites
*E*_ε_	Set of links on the topology in which every GEO is linked to each ground station
*E*_Ω_	Set of links on the fully meshed topology of the GEOs
*E*_τ_	Set of links on the topology in which every GEO is linked to each LEO
*R*(*t_i_*)	Set of key service requests in the time slot *t_i_*
*P*(*t_i_*)	Set of the rest of the secret keys of QKPs in the time slot *t_i_*
*F*_α_(*t_i_*)	Set of the rest of the time resources on the link between GEOs and ground stations
*F*_β_(*t_i_*)	Set of the rest of the time resources on the link between GEOs
*P*_d_	Set of links included in the QKD relaying path
*K*(*t_i_*)	Set of QKD key rates on the link in the time slot *t_i_*
*K*_s_	Set of number of secret keys required for the key service request
*r*(*s_r_, d_r_, k_r_, t_i_*)	Key service request generated in the time slot *t_i_*, *r*(*s, d, k, t_i_*) ∈ *R*(*t_i_*)
*s_r_*	Source node of the key service request *r*
*d_r_*	Destination node of the key service request *r*
*k_r_*	Number of secret keys required for the key service request *r*
*p*(*m*, *n, t_i_*)	Rest of the secret keys of QKPs on the link (*m*, *n*) in the time slot *t_i_*, *p*(*m*, *n, t_i_*) ∈ *P*(*t_i_*)
*f*_α_(*m*, *n, t_i_*)	Rest of the time resources on the link (*m*, *n*) between GEOs and ground stations, *f*_α_(*m*, *n, t_i_*) ∈ *F*_α_(*t_i_*)
*f*_β_(*m*, *n, t_i_*)	Rest of the time resources on the link (*m*, *n*) between GEOs, *f*_β_(*m*, *n, t_i_*) ∈ *F*_β_(*t_i_*)
*v*(*m*, *n*, *t_i_*)	QKD key rate (fixed in a time slot *t_i_*) on the link (*m*, *n*) in the time slot *t_i_*, *v*(*m*, *n*, *t_i_*) ∈ *K*(*t_i_*)
*c*(*m, n*, *t_i_*)	Boolean variable that is equal to 1 if the link (*m*, *n*) is established with the quantum channel in the time slot *t_i_*; otherwise, it is equal to 0
*l*_g_	Limitation of the number of quantum channels established from one LEO to the ground stations
*l*_b_	Limitation of the number of quantum channels established from one LEO to other LEOs
*l*_h_	Limitation of the number of quantum channels established from one GEO to LEOs/ground stations
Δ	Interval of a time slot of the physical topology
*N*_r_(*t_i_*)	The number of successful key service requests in the time slot *t_i_*
*N*_h_(*t_i_*)	The total number of relaying hops in the time slot *t_i_*
*ϕ*_l_(*t_i_*)	Number of secret keys produced by LEOs in the time slot *t_i_*
*ϕ*_g_(*t_i_*)	Number of secret keys produced by GEOs in the time slot *t_i_*
*ϖ*	Average keys for one request

## Data Availability

Not applicable.

## References

[1] Yang Z., Zolanvari M., Jain R. (2023). A Survey of Important Issues in Quantum Computing and Communications. IEEE Commun. Surv. Tutor..

[2] Gill S.S., Kumar A., Singh H., Singh M., Kaur K., Usman M., Buyya R. (2022). Quantum computing: A taxonomy, systematic review and future directions. Softw.-Pract. Exp..

[3] Lo H.-K., Curty M., Tamaki K. (2014). Secure quantum key distribution. Nat. Photonics.

[4] Pirandola S., Andersen U.L., Banchi L., Berta M., Bunandar D., Colbeck R., Englund D., Gehring T., Lupo C., Ottaviani C. (2020). Advances in quantum cryptography. Adv. Opt. Photonics.

[5] Lo H.-K., Chau H.F. (1999). Unconditional Security of Quantum Key Distribution over Arbitrarily Long Distances. Science.

[6] Scarani V., Bechmann-Pasquinucci H., Cerf N.J., Dušek M., Lütkenhaus N., Peev M. (2009). The security of practical quantum key distribution. Rev. Mod. Phys..

[7] Bennett C.H., Brassard G. Quantum Cryptography: Public Key Distribution and Coin Tossing. Proceedings of the IEEE International Conference on Computers, Systems and Signal Processing.

[8] Li W., Zhang L., Tan H., Lu Y., Liao S.-K., Huang J., Li H., Wang Z., Mao H.-K., Yan B. (2023). High-rate quantum key distribution exceeding 110 Mb s^−1^. Nat. Photonics.

[9] Liu Y., Zhang W.-J., Jiang C., Chen J.-P., Zhang C., Pan W.-X., Ma D., Dong H., Xiong J.-M., Zhang C.-J. (2023). Experimental Twin-Field Quantum Key Distribution over 1000 km Fiber Distance. Phys. Rev. Lett..

[10] Vallone G., Bacco D., Dequal D., Gaiarin S., Luceri V., Bianco G., Villoresi P. (2015). Experimental Satellite Quantum Communications. Phys. Rev. Lett..

[11] Liao S.-K., Cai W.-Q., Liu W.-Y., Zhang L., Li Y., Ren J.-G., Yin J., Shen Q., Cao Y., Li Z.-P. (2017). Satellite-to-ground quantum key distribution. Nature.

[12] Liao S.-K., Cai W.-Q., Handsteiner J., Liu B., Yin J., Zhang L., Rauch D., Fink M., Ren J.-G., Liu W.-Y. (2018). Satellite-Relayed Intercontinental Quantum Network. Phys. Rev. Lett..

[13] Simon C. (2017). Towards a global quantum network. Nat. Photonics.

[14] Bedington R., Arrazola J.M., Ling A. (2017). Progress in satellite quantum key distribution. Npj Quantum Inf..

[15] Vergoossen T., Loarte S., Bedington R., Kuiper H., Ling A. (2020). Modelling of satellite constellations for trusted node QKD networks. Acta Astronaut..

[16] Calderaro L., Agnesi C., Dequal D., Vedovato F., Schiavon M., Santamato A., Luceri V., Bianco G., Vallone G., Villoresi P. (2018). Towards quantum communication from global navigation satellite system. Quantum Sci. Technol..

[17] Cao Y., Zhao Y., Wang Q., Zhang J., Ng S.X., Hanzo L. (2022). The Evolution of Quantum Key Distribution Networks: On the Road to the Qinternet. IEEE Commun. Surv. Tutorials.

[18] Hosseinidehaj N., Babar Z., Malaney R., Ng S.X., Hanzo L. (2018). Satellite-Based Continuous-Variable Quantum Communications: State-of-the-Art and a Predictive Outlook. IEEE Commun. Surv. Tutor..

[19] Khan I., Heim B., Neuzner A., Marquardt C. (2018). Satellite-based QKD. Opt. Photonics News.

[20] Takenaka H., Carrasco-Casado A., Fujiwara M., Kitamura M., Sasaki M., Toyoshima M. (2017). Satellite-to-ground quantum-limited communication using a 50-kg-class microsatellite. Nat. Photonics.

[21] Grieve J.A., Bedington R., Tang Z., Chandrasekara R.C., Ling A. (2018). SpooQySats: CubeSats to demonstrate quantum key distribution technologies. Acta Astronaut..

[22] Liao S.-K., Yong H.-L., Liu C., Shentu G.-L., Li D.-D., Lin J., Dai H., Zhao S.-Q., Li B., Guan J.-Y. (2017). Long-distance free-space quantum key distribution in daylight towards inter-satellite communication. Nat. Photonics.

[23] Gündoğan M., Sidhu J.S., Oi D.K.L., Krutzik M. (2023). Time-delayed single quantum repeater node for global quantum communications with a single satellite. arXiv.

[24] De Grossi F., Alberico S., Circi C. (2023). Orbit Design of Satellite Quantum Key Distribution Constellations in different Ground Stations Networks. Adv. Space Res..

[25] He X., Li L., Han D., Zhao Y., Nag A., Wang W., Wang H., Cao Y., Zhang J. (2022). Routing and secret key assignment for secure multicast services in quantum satellite networks. J. Opt. Commun. Netw..

[26] Wang J., Chen H., Zhu Z. (2021). Modeling research of satellite-to-ground quantum key distribution constellations. Acta Astronaut..

[27] Günthner K., Khan I., Elser D., Stiller B., Bayraktar Ö., Müller C.R., Saucke K., Tröndle D., Heine F., Seel S. (2017). Quantum-limited measurements of optical signals from a geostationary satellite. Optica.

[28] Huang D., Zhao Y., Yang T., Rahman S., Yu X., He X., Zhang J. (2020). Quantum Key Distribution Over Double-Layer Quantum Satellite Networks. IEEE Access.

[29] Aguado A., Hugues-Salas E., Haigh P.A., Marhuenda J., Price A.B., Sibson P., Kennard J.E., Erven C., Rarity J.G., Thompson M.G. (2017). Secure NFV Orchestration Over an SDN-Controlled Optical Network with Time-Shared Quantum Key Distribution Resources. J. Light. Technol..

[30] Picchi R., Chiti F., Fantacci R., Pierucci L. (2020). Towards Quantum Satellite Internetworking: A Software-Defined Networking Perspective. IEEE Access.

[31] Su Y., Liu Y., Zhou Y., Yuan J., Cao H., Shi J. (2019). Broadband LEO Satellite Communications: Architectures and Key Technologies. IEEE Wirel. Commun..

